# Interaction network analysis of the six game complexes in high-level volleyball through the use of Eigenvector Centrality

**DOI:** 10.1371/journal.pone.0203348

**Published:** 2018-09-11

**Authors:** Lorenzo Laporta, José Afonso, Isabel Mesquita

**Affiliations:** 1 Centre for Research, Formation, Innovation and Intervention in Sport, Porto, Portugal; 2 Faculty of Sport, University of Porto, Porto, Portugal; Southwest University, CHINA

## Abstract

Social Network Analysis establishes a network system and provides information about the relationships (edges) between system components (nodes). Although nodes usually correspond to actors within the network (e.g., the players), it is possible to stipulate *game actions* as nodes, thus creating a network of the flow of game actions. In this study, Eigenvector Centrality (a form of weighted centrality that considers *n-*order connections) was used to identify differences in the centrality of distinct game actions within each of the six game complexes of volleyball. Thirteen matches (46 sets, 2,049 rallies) of the final round of the 2015 FIVB's World Grand Prix (Women) were analyzed. Results showed that analyzing actions as actors (i.e., nodes) offers a clear and comprehensive understanding of game flow and poses an interesting alternative to mainstream research where players are considered nodes. Functional differences between the six game complexes were highlighted, denoting the validity of such division. Out-of-system playing (i.e., having to set the attack under non-ideal conditions, e.g., in KI, KII, KIII and KIV), emerged as a regularity of the game and should be translated into the training process.

## Introduction

The need for a systemic analysis of performance has been proposed extensively (e.g., [1–3]). In this vein, performance analysis (PA) is conducted to establish an ecological view via the understanding of the relationships between variables within a system [1]. Systemic approaches to PA treat systems as wholes, albeit composed of interacting independent parts [2], and as entities capable of self-organizing to produce non-linear patterns. Team sports (TS) fall into this conceptual framework because they are characterized by interconnected systems that change their state over time through self-organization properties [3]. It can be difficult to access the inherent complexity of systems, although some promising methods have emerged to address this issue.

Among such methods, Graph Theory (GT) provides a useful topological analysis of a complex system that offers quantitative insights into systemic behavior [4], which may then be interpreted qualitatively. Mathematical GT has been the key to generating advanced quantitative methods for Social Network Analysis (SNA) [5]. Making use of methods derived from GT and sociometry, SNA offers a systemic view of the game [6, 7] and establishes a global network of the system through the study of interconnections (edges) among sets of actors (nodes) [8]. As such, SNA highlights the structural relationship between actors and reveals global network features, as well as individual network positions and subgroups within a whole network, which are the most important information transmitters [9]. Among others, centrality–which reveals the network position of individual actors or elements–is a key concept of SNA (see e.g. [10]).

Although SNA has existed for some time [11, 12], it has only been applied to sport relatively recently, which is evident in the systematic review published by Wäsche and colleagues [13]. From this work, Wäsche and colleagues proposed a six-dimension conceptual typology of SNA application that offers a renewed understanding and systematization of this new field research field. While two of these dimensions (i.e., competition networks and interaction networks) are specific to the sport, the other four (i.e. inter-organizational network, intra-organizational networks, affiliation networks, and social environments) are related to other social contexts. Competition networks (inter-event) examine sport outcomes and results by the identification of structural patterns and relative performances of athletes or sports teams. Interaction networks, on the other hand, focus on the interactions between players within a team, thus highlighting the relationships between game actions and how such interconnectivity can influence effectiveness.

As showed by Wäsche et al. [13], competition networks are much more widely studied than interaction networks, which reinforces the value of researchers exploring the latter. Here, the focus of research has been on the interactions between players (nodes) in different game phases through their passes (edges). For example, Gama et al. [6] examined attack patterns in soccer via the individual actions of key players and their influence on team performance in the Portuguese Football Premier League; their personalized metric was similar to degree centrality. Clemente et al. [14] assessed the intra-team tactical behavior in basketball attack organization using distinct centrality metrics of network (degree prestige, degree centrality). Duch et al. [15] analyzed the performance of individual players, and of teams, in the 2008 European Cup soccer tournament and concluded that flow centrality provides a powerful objective quantification of individual and team performance.

Such contributions have provided useful insights, but interaction networks such merit a broader body of research that incorporates the relational perspective proposed by Emirbayer [16] and Elster [17]. Additionally, SNA also offers the possibility of defining action variables as nodes [18, 19], and their relationships as edges. This perspective brings a departure from most current research and provides different avenues for understanding game flow. This action-centered approach (as opposed to a player-centered approach) might constitute a more suitable pathway towards an understanding of the functional specificities of each game phase (in volleyball, the technical term is game complex), and thereby paving the way to better pedagogical models for teaching the game and training models that are more coherent with the demands of the game [19].

The centrality of a variable is usually determined by the absolute number of its direct connections (e.g., Degree Centrality [10]), which might provide an analysis that neglects indirect connections. Eigenvector Centrality, in comparison, weights the indirect connections in addition to direct connections (e.g., second- or third-order links between nodes) [10, 20], and therefore provides a more complete overview of the role of each node within a systemic perspective. Until now, centrality measures such as Eigenvector Centrality have mainly been adopted by sociometric approaches and quantitative network studies of ethnography [5, 21]. However, to our knowledge, this metric is still underused in performance analysis of sports [19, 22, 23]. One exception is a study by Sasaki et al. [24], which used Network Centrality Analysis (Density and Eigenvector) to determine the tactical leader of high-level rugby teams (2015 Rugby World Cup) and to analyze the impact of defensive actions on the outcome of the game. This revealed the existence of decisive relational structures where the highest turnover performance would contribute to the winning game, and that certain individuals play key roles in the game (e.g., “fly-half"). Because game events are likely to produce direct as well as indirect consequences, the application of Eigenvector Centrality in high-performance sport settings needs to be further explored [25].

Volleyball is a team sport composed of six functionally distinct but interconnected game complexes (Ks). The six game complexes are as follows (based on [18, 19, 26]): Complex 0 (K0) or serve; Complex I (KI) or side-out; Complex II (KII) or side-out transition; Complex III (KIII) or transition of transition; Complex IV (KIV) or attack coverage; and Complex V (KV) or Freeball and Downball. Despite the rationale behind the theoretical compartmentalization of volleyball into six functionally distinct game complexes, research has focused mainly on KI and KII (e.g. [27, 28]), usually incorporating K0, KIII, KIV and KV into KII. This combining of complexes has produced results that might be misleading because important inter-complex differences are likely to be averaged out. Although a number of investigations have focused on the characteristics of subsets of the six game complexes [29, 30], analyzing the game without considering all the complexes may limit the ability to acquire an in-depth understanding of the game [30]. Here, we propose to address this issue in women’s volleyball, a decision made because women’s volleyball is less well studied than men’s volleyball [31].

The application of SNA to volleyball is still in a preliminary phase, and Eigenvector Centrality has seldom been applied. Indeed, to date just three studies have utilized Eigenvector Centrality [18, 19, 23]. These studies have investigated complexes I, II and III in the group stage of the women's 2015 World Grand Prix [23] and the men's 2015 World Cup [22], and also complexes IV and V in the group stage of the women's 2015 World Grand Prix [19]. More research is, however, required to fully explore the contributions of Eigenvector Centrality in sports. This is particularly true for women’s volleyball because it is less well studied than men’s volleyball [31]. Moreover, studies on interaction networks have not used Eigenvector Centrality [7, 15, 32, 33], and thus an opportunity exists for this to be explored.

Our aim, therefore, was to examine the functional differences between the six game complexes in high-level women’s volleyball from the perspective of SNA, and specifically using the insights offered by Eigenvector Centrality. Here, we will use an action-centered approach and consider game actions as nodes to establish an interaction network [13]. We assessed all six game complexes because we expected each to have different characteristics and weights for each variable, particularly setting conditions. More specifically, we anticipated that the weights for the most critical game actions (i.e., Setting Conditions, as they represent the link between the first and final contact with the ball) would be different for each complex, albeit with a predominance of Setting Condition C (i.e. poor conditions for setting the attack). We also hypothesized that the patterns for each complex would depend on the interconnectivity with previous complexes. Here, we specifically anticipated that block opposition would be enhanced (i.e., more blockers opposing the attack) when playing in KII and KIII, and impaired when playing in KIV and KV.

## Material and methods

### Sample

Thirteen matches of the final round of the 2015 FIVB World Grand Prix (one of the main world volleyball competitions for women) between the national teams of Brazil (4 matches), China (5 matches), Japan (4 matches), Italy (4 matches), Russia (4 matches) and the USA (5 matches) were observed. Thus, a balanced number of matches for each team was observed. We analyzed a total of 46 sets (8 three-set matches, 3 four-set matches, and 2 five-set matches), which corresponds to 2,000 plays or rallies, including 2,017 ball possessions related to K0, 1,800 to KI, 1,423 to KII, 1,204 to KIII, 258 to KIV, and 273 to KV. This resulted in the production of a network with 125 nodes and 1,865 edges. The Ethics Committee at the Centre of Research, Education, Innovation and Intervention in Sport, University of Porto, provided institutional approval for this study (CEFADE 16.2017).

### Measures

**Fig 1:**
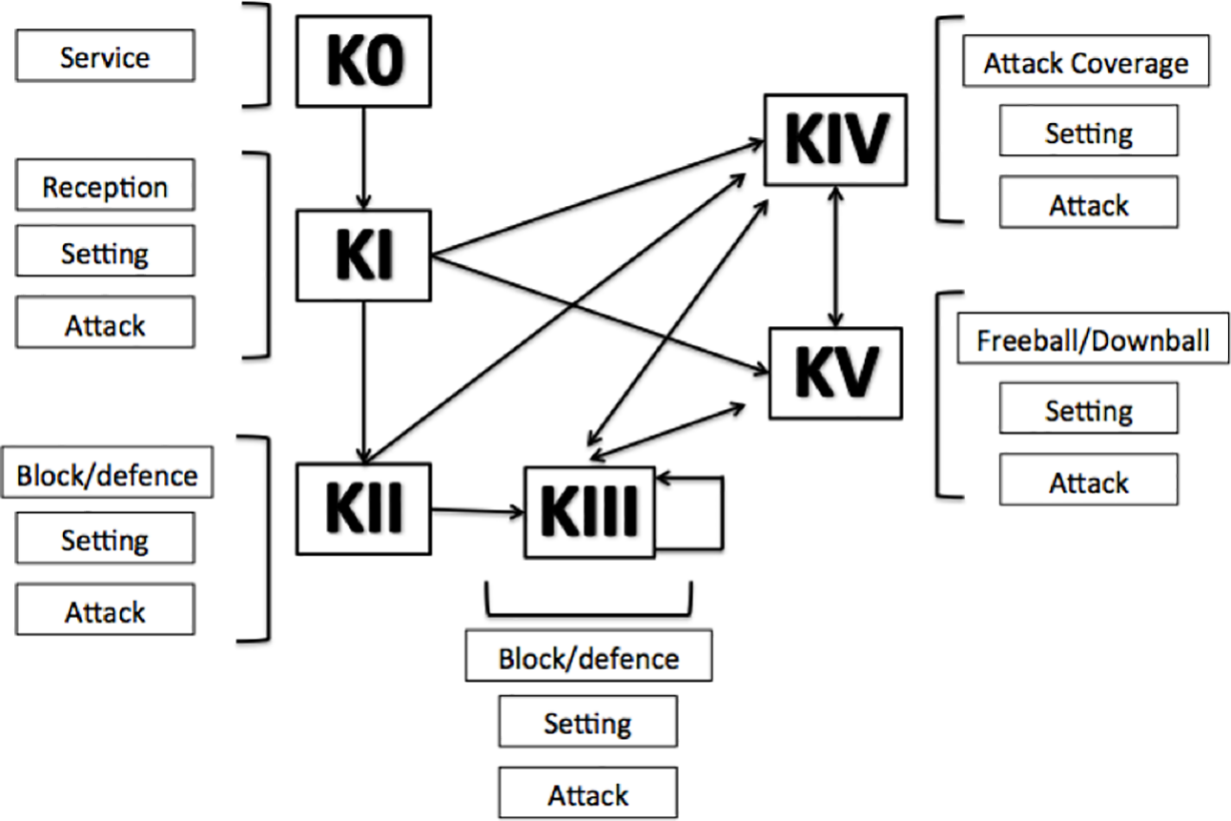
The six game complexes in volleyball.

The game of volleyball can be divided in phases or subsystems, usually termed *game complexes* in the specialized literature [26]. Each game complex displays distinctive features and sequences of occurrence. Six *Game Complexes* (K’s) were considered and are synthesized in Fig 1: Complex 0 (K0) or serve; Complex I (KI) or side-out; Complex II (KII) or side-out transition; Complex III (KIII) or transition of transition; Complex IV (KIV) or attack coverage; and Complex V (KV) or Freeball and Downball (based on [18, 19, 26]).

**Fig 2:**
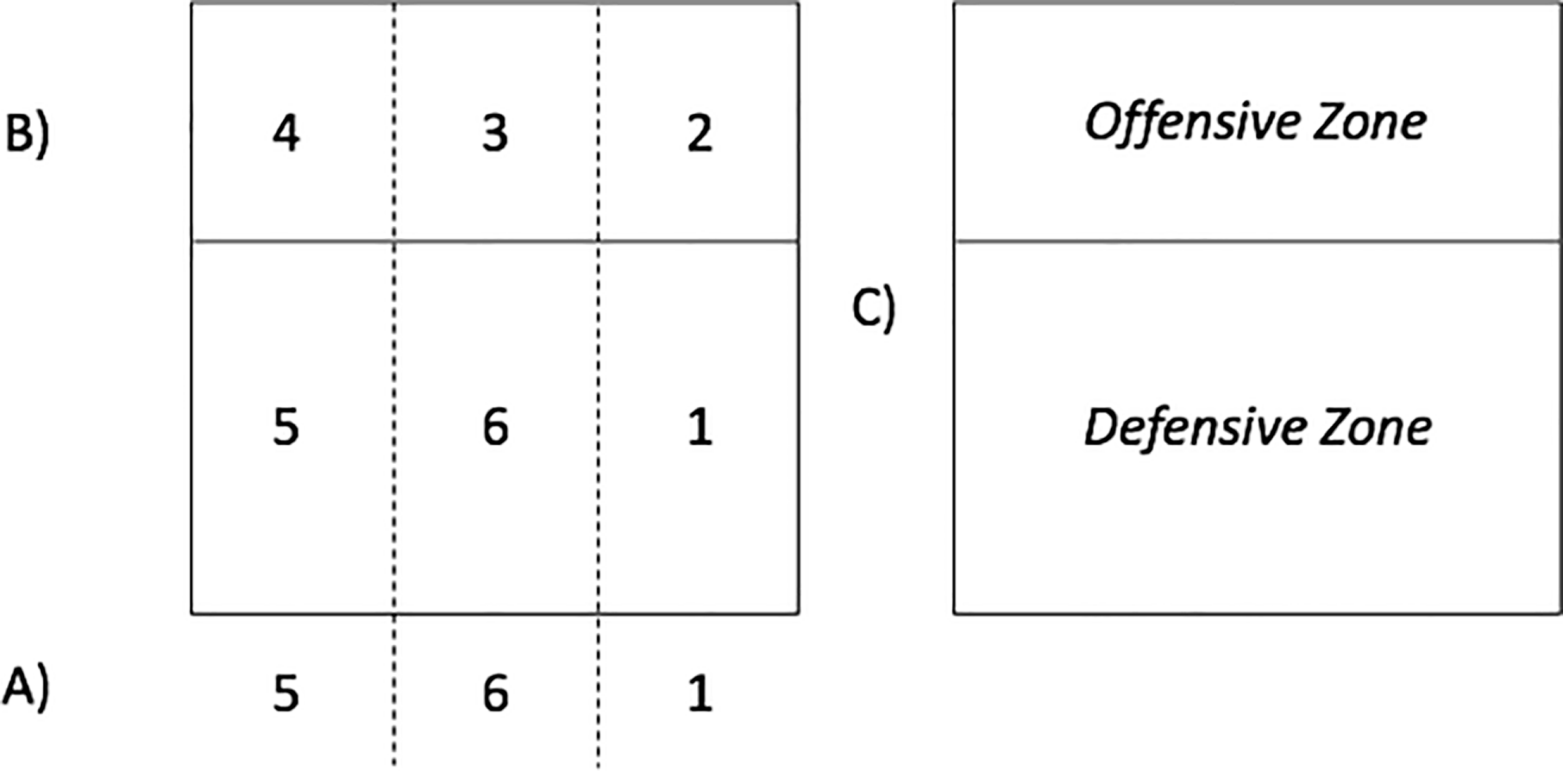
Variables illustration of initial position of server, First Contact, Attack Zone and Target Zone in KV. (A) Initial Position of the server; (B) First contact and Attack Zone; and (C) Target Zone in KV.

The *Initial Position of the Server* was based on Quiroga et al. [34]: Zones 1, 6 or 5; see Fig 2A. *Serve Type* was adapted from Costa et al. [31] Quiroga et al. [34]: float jump serve (i.e., without ball rotation); jump serve (i.e., with ball rotation); and standing serve (i.e., without jumping and including both the float and topspin standing serves; their reduced occurrence in high-level volleyball justifies their grouping into a single category).

The *Zone of First Contact* can occur in the form of reception or defense and was stipulated following the six official zones according to the FIVB rules (see Fig 2B). In the case of defense, we added the ‘Others’ (OT) zone. This corresponds to the area where the athlete can retrieve balls outside the bounds of the court after having touched the block. In serve-reception, this category is not required, as a previous contact with the block is not possible.

*Setting Conditions* were adapted from Marcelino et al. [28], Laporta et al. [30], Afonso and Mesquita [35], and refer to the attack options available to a setter before setting the attack: A—the setter has all the attack options available, and therefore the ideal setting conditions; B–the setter can deploy quick attacks, but some attack options are not possible (e.g., crossing of players); C–the setter can only use high sets, and thus it is considered as out-of-system playing.

The *Attack Zone* made use of the six official zones determined by FIVB rules (see the Fig 2A). Attack Tempo was simplified from Afonso and Mesquita [36] and Costa et al. [31], and refers to the synchronization between the setter and the attacker: a) in tempo 1 the attacker jumps before or at the same time as the set; b) in tempo 2 the attacker performs two steps after the set; and c) in tempo 3 the attacker waits for the ball to ascend and then performs a three- or more-steps approach.

*Block Opposition* was adapted from Afonso and Mesquita [35], and considered the following categories: no-block (B0); single or individual block (B1); double block (B2); and triple block (B3). The *Number of Available Players Before Attack Coverage* was adapted from Laporta et al. [30], Laporta et al. [37] and refers to the players that were available to attack before an attack coverage occurring. The *Number of Coverage Lines* consists of imaginary curved lines (progressing from the net to the endline) established by the players that were in the defense position in the moment of the attack.

*Freeball* represents the team organization for a ball that will have to be returned softly by the opponent due to poor conditions for performing the third contact [38]; *Downball* occurs when it is unfavorable for a player to attack, but they still perform a standing spike [39]. The *Target Zone in KV* (see Fig 2C) is the area of the court in which the ball was dropped. It can be categorized as an offensive or defensive zone according to official FIVB rules (i.e., from the 3-meter line until the net or behind the 3-meter line). The analyzed variables are summarized in S1 Table.

### Data collection

The matches were viewed using the websites *laola*.*tv* and *youtube*.*com*. All matches were available in high-definition (1080p) and recorded with a moving lateral view of the court (i.e., broadly aligned with the net and with a moving angle), which was suitable for the variables we intended to observe in this investigation. Indeed, the variables were selected considering these specifications. Likewise, we chose specific instruments to analyze our variables of interest considering the characteristics of the video footage.

### Data worksheet

The data were input into an analysis worksheet created with Microsoft^Ⓡ^ Excel^Ⓡ^ 2017 (Microsoft Office Professional 365 Version 15.30, E.U.A.) using the macro function to instantly catalogue the required codes in the appropriate cells.

### Training protocol for the observers

For the observer’s training protocol we expanded upon the methodological concerns outlined by Araújo et al. [40]. Three observers, each with a master's degree in the area of volleyball training and with extensive experience as a volleyball coach (i.e., more than five years as coaches and with victories in national championships in their résumé), were trained to use the instrument for a period of three months. Two reliability tests were performed in this period (the first after two months of testing the instrument, another three months after) to ensure consistency and to allow for any necessary adjustments to the variables and categories of the final instrument. Over the three months of training, weekly meetings were held for instrument explanations and clarifications, discussion of emerging problems, and a joint analysis of different matches (not used in the current investigation) [41].

As the analysis is designed to apply to both women and men’s volleyball, the first reliability testing comprised an analysis of 217 actions from a high-level men's match (final match to qualify for the 2015 FIVB Volleyball World League Pool E, totaling 5 sets). Four of the 13 variables obtained Kappa values below 0.75. This led to an in-depth discussion about these variables and their categories, which we then redefined and improved to increase clarity and the likelihood of more homogeneous recordings.

After further training meetings (analyzing both men’s and women’s matches), a second reliability test of the instrument was performed, this time using a high-level women’s match (play-off match of 2014/2015 Turkish Women's Volleyball League, totaling 5 sets), and a total of 209 actions. This match was from a different competition to that considered in our study) in order to avoid bias when moving towards analysis of the target competition. While reliability improved, one variable (Type of Reception Line) still presented values below the expected 0.75. Therefore, after critical discussions, the researchers decided to remove this variable from the study. The final analysis worksheet thus included the 11 variables described in S1 Table.

We conducted the final assessment of data reliability measurement with 415 actions from two high-level matches (final phase of the 2015 World League and 2015 World Grand Prix, totaling 10 sets). All variables presented Cohen's Kappa values above 0.75, as suggested in the literature [42].

### Data analysis

After being inserted, data were examined using SPSS^Ⓡ^ for Mac (Version 24, IBM^®^, E.U.A.). A descriptive analysis was conducted to ensure data quality (verify input errors, data frequency and others). A calculation of Eigenvector Centrality was then performed using Gephi 0.9.1 for Mac (MacRoman, France). Nodes were placed at the periphery of the network so that all interactions could be clearly visualized.

The contrast of node size and color were perfected to reflect the magnitude of their Eigenvector values. Node size was manipulated using the intrinsic units provided by the software, which were specified between 300 (minimum) to 1,500 (maximum). These values are a measure of arbitrary, relative units, where the value (node size) determines the degree of visual contrast between variables according to the different Eigenvector values.

Edges were also depicted with a variable thickness in order to better reflect Eigenvector values. Although nodes reflect the weight of both direct and indirect connections, edges provide a measure of direct connections only. Therefore, thicker edges correspond to a greater number of connections between two nodes. Edges are defined in units, i.e., number of connections. A direct connection between two variables is established if they are simultaneous or consecutive. For example, Attack Tempo occurs simultaneously with Attack Zone, so some category of Attack Tempo will always connect to some category of Attack Zone. Also, Attack Tempo is preceded by Setting Conditions and followed by Block Opposition. Therefore, it also establishes direct connections with these two variables. However, there are no direct connections between Attack Tempo and Zone of First Contact, as they do not follow consecutively. Nonetheless, Eigenvector Centrality calculates the weight of indirect connections, such as the following: Zone of First Contact–Setting Conditions–Attack Tempo.

### Data reliability

After data collection was completed, inter-observer reliability was assessed with 10% of the total sample (a total of 210 randomly chosen actions) as suggested in the literature [42]. A calculation of Cohen's Kappa provided values ranging between 0.80 and 1, which are above the threshold of 0.75 proposed by Tabachnick and Fidell [43]. Intra-observer reliability analysis was conducted with the same 210 actions approximately two months after the first observations. All variables achieved values of Cohen’s Kappa between 0.791 and 1, again surpassing the minimum accepted threshold of 0.75.

## Results

**Fig 3:**
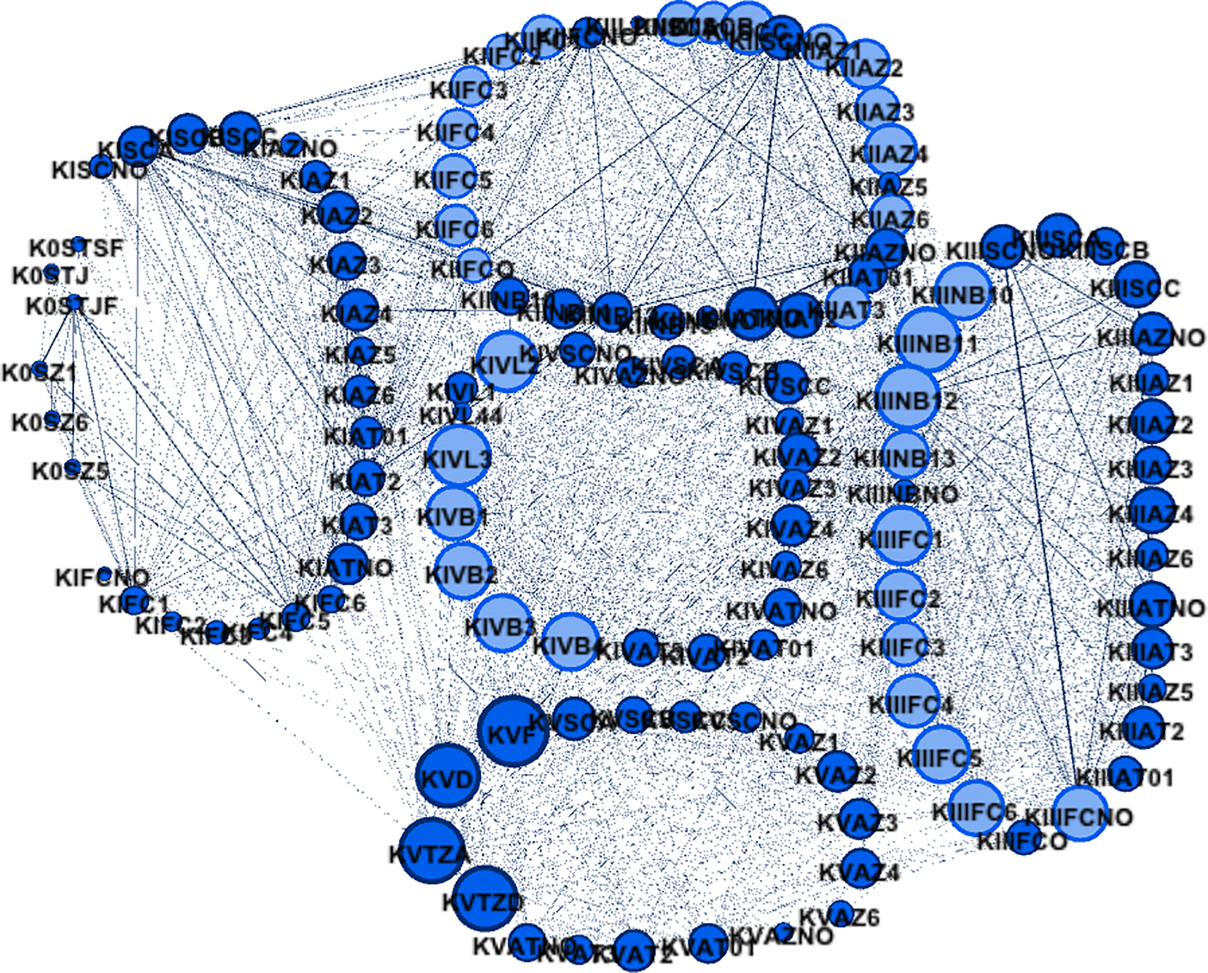
Network with Eigenvector Centrality for all complexes. Terminology: in each node, codes are represented by the name of complex (e.g., KII), followed by the variable and its category (e.g., KVFCZ6 indicates that the action occurred in complex V, the variable in question was Zone of First Contact, and the category is Zone 6). Codes for the different variables: IPS–Initial Position of the Serve; ST–Serve Type (Jump, Jump-Float and Standing-Float); FC–Zone of First Contact; SC Setting Condition; AZ–Attack Zone; AT–Attack Tempo; BO–Block Opposition; KIVB–Number of Available Player Before of Attack Coverage; KIVL–Number of Coverage Lines; KVD and KVF–Downball and Freeball; KVTZ–Target Zone in KV (Attack or Defense Zone).

A global network of within-complex and between-complex interactions was established (Fig 3) using Eigenvector Centrality to provide a map of interactions.

A visual inspection of the network revealed the existence of functional distinctions between game complexes. Quantitative values of Eigenvector Centrality for each game complex are exhibited in S2–S7 Tables.

Eigenvector values for the variables belonging to Complex I are presented in S3 Table, highlighting the high values for Setting Condition C, Attack Zones 2 and 4 and Attack Tempo 2 and 3.

S4 Table presents the variables belonging to Complex I. Note the high centrality values for Setting Condition C, Attack Zone 4, Attack Tempo 2 and 3, and Single and Double-block.

Eigenvector values for the KIII variables are shown in S5 Table. Setting Condition C and Attack Tempo 2 presented high values in their categories. In KI, the zones with higher Eigenvector values were Zone 1 and 5, followed by Zone 6, whereas in KII, Zones 5, 1 and 6 showed the highest values. The same regularity was observed in KIII.

Block Opposition had similar values for KII and KIII, with Double Block presenting the highest values, followed by Single Block. In KIII the Triple Block obtained an Eigenvector value superior to that of KII.

The Eigenvector values of the variables belonging to Complex IV are presented in S6 Table: note the high values for Setting Condition C, Attack Zones 2 and 4 and Attack Tempo 2 and 3.

S7 Table shows the Eigenvector values of the complex V variables, where Setting Conditions A, Attack Tempo 1 and 2 revealed a higher value of centrality. For Setting Conditions, KI exhibited the highest Eigenvector value for Setting Condition C, followed by Setting Conditions A and B. In KII, Setting Condition C also had a higher centrality value, with lower values for Setting Conditions A and B. KIII followed the same trend of Setting Condition C having the highest value, although Setting Condition A had a larger value than Setting Condition B. KIV was defined by higher values of Eigenvector Centrality compared to the other complexes, and in it Setting Condition C had the highest Eigenvector value, well above the values for Setting Conditions A and B. Finally, KV was the only game complex where Setting Condition A had the highest value, followed by Setting Conditions B. For this complex, Setting Condition C had the lowest centrality value.

In KI, the Attack Zones with the greatest Eigenvector centrality were Zones 2, 4 and Zone 3. In KII, Zones 2 and 4 had the highest centrality values, followed by Zone 1. Attack Zone 4 was the most central in Complex III, followed by Zones 2 and 3. In KIV, Zone 2 and 4 presented the highest values, while the centrality of other Attack Zones fell below 0.3. In KV, in addition to Zones 2 and 4, Zone 3 also presented a high Eigenvector value.

The variable Attack Tempo had similar values for all categories in KI, although Attack Tempos 2 and 3 had the highest Eigenvector values, followed by Attack Tempo 1. In KII, Attack Tempos 2 and 3 had the highest Eigenvector values. In KIII, Tempo 2 presented the highest value, followed by Tempos 3 and 1. In KIV Tempo 2 had the highest value, followed by Tempo 3. In KV, the most central categories were Tempos 2 and 1, with Tempo 3 exhibiting the lowest value of this complex.

## Discussion

The aim of the present study was to conduct a systemic analysis of high-level women’s volleyball, with special consideration of the game actions comprising its six interconnected game complexes. Our rationale for this was based on the six-dimension conceptual typology of Social Network Analysis (SNA) applied to sport Wäsche et al. [13], which establishes an interaction network to examine the degree of interconnectivity and specificity of the different game complexes. In order to establish the quantitative and topological relationships between game variables across the six complexes we used SNA and Eigenvector Centrality. This metric provides the opportunity to access not only the direct connections between game actions, but also the indirect connections within each complex and between complexes. Another novel aspect of this research was the analysis of game actions as nodes, while previous research has applied player-centered approaches.

*Preferential attachments* [7] from indirect and direct connections were identified, and these highlight features of the game dynamics within each complex and between complexes. The identification of such preferential attachments within a small-world network from an interaction network (intra-event) creates the possibility for identifying the critical game actions that impact more on the game flow, and therefore on the performance. Moreover, this paper answered the call by Passos et al. [7] for more research that measures the temporal and spatial distribution of high frequency nodes pertaining to interactions in team sports. Here, we have accomplished this with a relevant sample of 2,017 ball possessions in a total of 46 sets, with 125 nodes and 1865 edges.

We hypothesized that some game actions would have different weights in distinct game complexes, and would thus have varied impacts on game flow, i.e. on the patterns and configurations of the game dynamics. By using Eigenvector Centrality it was evident that there were *preferential attachments* [7] that explicitly impacted on the game dynamics. For instance, playing out-of-system (strongly related to Setting Conditions, Attack Tempo and Zone) emerged as a central game pattern in several complexes. Setting the attack under non-ideal conditions (i.e. Setting Condition C) had a central role in almost all complexes (I, II, III and IV). This suggests that in four of five game complexes (as K0 is an enclosed complex with only one action, i.e., the serve), setting out-of-system is more central than setting in-system (A), or marginally out-of-system (B), conditions. Although setting in-system is considered crucial for creating the best options for attack tempo and attack zone [44], our data emphasized that the use of non-ideal, out-of-system setting conditions is a regularity in high-level women’s volleyball. These findings are somewhat different to those of previous research using Eigenvector centrality in women’s volleyball. Hurst et al.[23] analyzed the first phase of the 2015 World Grand Prix and showed that setting conditions had roughly equivalent centrality values in complexes I and III, while setting out-of-system was only predominant in KII. Our study was applied to the final round of the 2015 World Grand Prix, which is likely to have more balanced matches because the team levels are highly equalized (after all, only the best of the best classify for this stage). These differences in findings might, therefore, depend on the competition stage, although more research is necessary to verify if this trend is consistent in other samples.

In this study, Eigenvector Centrality was shown to be a powerful tool for establishing the interconnectivity between game actions and their relative roles in each game complex (Setting Conditions, for example, establish the possibility for linking the first contact with the third contact in a functional manner). This requires a consideration of the interplay between all the game actions (i.e., the whole network), hence highlighting the clear advantage of adopting the SNA approach and Eigenvector metric. Similarly, we observed higher centrality values for slower attack tempos, and attacks at the extremities of the net, and it is possible that these are derived from the predominant out-of-system setting conditions. Although prior studies already devoted some attention to Setting Conditions (e.g., [45, 46], [47]), none considered the interactions among all the different game actions, i.e. there was no weighting of indirect connections. Such studies have also tended to analyze each condition was studied in a somewhat rigid manner, such as by considering specific zones of the court, instead of adopting a more flexible and functional analysis. They therefore do not correspond well to the reality and complexity of high-level games.

We anticipated that the interconnectivity with previous complexes would influence the patterns of following game complexes: block opposition, for instance, would be enhanced in KII and KII (i.e., more blockers opposing the attack in more unpredictable complexes) and impaired conditions in complexes with less uncertainty (i.e., KIV and KV). Our use of Eigenvector Centrality allowed us to identify some specificities of the game complexes, and the relationships each complex had with subsequent complexes. KII, for example, exhibited a greater unpredictability because it had consistent centrality values across the different attack zones and tempos. Such uncertain conditions then made it possible for KIII to be more predictable, evident by Attack Tempo 2 having a higher Eigenvector value than the Attack Tempos 1 and 3. In KV, there was greater Eigenvector Centrality for faster attack tempos (i.e., Tempos 2 and 1), coupled with a more even distribution of front row attack zones (i.e., Zones 2, 3 and 4). This implies that faster attack tempos favor the unbalancing of the block [35], which is supported in our study by higher centrality values for single and double blocks in KIII. The strong relationships between complexes and their subsequent game complexes are apparent, and this appeals to the utilization of methodologies that consider the interconnectivity between game actions and complexes, as is considered in the SNA approach.

Overall, the present study demonstrates the power of SNA in accessing the game ecology (i.e., in considering its dynamics and complexity) and for allowing the identification of game patterns that are context-dependent. Quantifying events in team sports while accounting for their interconnectivity is far from trivial. Moreover, the relevance of adopting an *action-centered approach*, as carried out in this study, is that it (a) provides a powerful and objective quantification of all actions considering both its direct and indirect linkages, and (b) delivers a deeper comprehension of the specificity and interconnectivity of game actions considering the game phases where they take place. In light of the shortcomings of previous studies on interaction networks, we believe the current research adds to the existing body of knowledge.

## Conclusions

In the present study, the use of Social Network Analysis provided important and weighted interaction patterns that respect the game’s ecology and that describe the specificities of, and relationships between, the six game complexes. Moreover, the use of Eigenvector metrics revealed which game events were most influential at each moment of the game by considering the indirect connections with other actions. Our data exhibited some game patterns that are not usually considered in the training process. Indeed, training is usually planned and developed considering the ideal conditions, such as setting the ball through the traditionally considered ideal condition for setting (i.e., Setting Condition A). However, as this study has shown, game patterns are diverse and playing under non-ideal conditions (such as Setting Conditions C and Attack Tempo 3) has a central role in almost all complexes. In this vein, coaches should consider this diversification and prepare teams to face the problems imposed by their opponent, that is, to act in real-game situations, or under non-ideal conditions, in order to optimize team preparation for competition [23, 48].

We also identified a strong relationship between each complex and its subsequent game complexes (e.g., KII with KIII). This suggests that training should consider this complex dependence, such as by analyzing the game scenarios and their probability of occurrence as a result of the actions performed in the previous complex. Prior studies on interaction networks have not used Eigenvector Centrality [7, 15, 32, 33]. Our study thus represents a step forward in considering both direct and indirect connections and delivers a more refined view of an interaction network, which we consider relevant for the understanding of the complex and dynamic nature of team sports.

One limitation of the present investigation is that the tactical systems used by each team, the characteristics of their players, and other situational constraints were not considered. However, the use of Eigenvector Centrality, the consideration of six functional game complexes, and especially the classification of game actions as nodes, has provided a novel approach to interaction networks.

Future studies using Social Network Analysis could make greater use of Eigenvector Centrality from a relational event perspective [49], such as by incorporating game constraints (e.g., players and situational cues) that impact upon the match dynamics. Here, it will also be of remarkable value to explore the game actions performed by players according to their functional specialization in the game [50], and situational variables such as match status [51], quality of opposition [52] and moment of the game [53], among others.

## Supporting information

S1 Table Summary of variables and occurrence in the game. (DOCX)

S2 Table Eigenvector Centrality values for Complex 0. (DOCX)

S3 Table Eigenvector Centrality values for Complex I. (DOCX)

S4 Table Eigenvector Centrality values for Complex II. (DOCX)

S5 Table Eigenvector Centrality values for Complex III. (DOCX)

S6 Table Eigenvector Centrality values for Complex IV. (DOCX)

S7 Table Eigenvector Centrality values for Complex V. (DOCX)

S1 File Gephi Data Base with Codes for the different variables: IPS–Initial Position of the Serve; ST–Serve Type (Jump, Jump-Float and Standing-Float); FC–Zone of First Contact; SC Setting Condition; AZ–Attack Zone; AT–Attack Tempo; BO–Block Opposition; KIVB–Number of Available Player Before of Attack Coverage; KIVL–Number of Coverage Lines; KVD and KVF–Downball and Freeball; KVTZ–Target Zone in KV (Attack or Defense Zone). (ZIP)

## References

[1] McGarry T, Anderson D, Wallace S, Hughes M, Franks I. Sport competition as a dynamical self-organizing system. Journal of Sports Sciences. 2002;20(10):771–81. 10.1080/026404102320675620 12363294

[2] Stein I. Systems theory, science, and social work: Scarecrow Press; 1974.

[3] Davids K. Athletes and sports teams as complex adaptive system: A review of implications for learning design.[Atletas y equipos deportivos como sistemas adaptativos complejos: Una revision de las Implicaciones para el diseño del aprendizaje]. RICYDE Revista Internacional de Ciencias del Deporte. 2015;11(39):48–61.

[4] Bondy JA, Murty USR. Graph theory with applications: Citeseer; 1976.

[5] Freeman L. The development of social network analysis: A Study in the Sociology of Science. North Charleston, South Carolina: BookSurge, LLC; 2004 10.1631/jzus.2004.1028

[6] Gama J, Passos P, Davids K, Relvas H, Ribeiro J, Vaz V, et al Network analysis and intra-team activity in attacking phases of professional football. International Journal of Performance Analysis in Sport. 2014;14(3):692–708.

[7] Passos P, Davids K, Araujo D, Paz N, Minguéns J, Mendes J. Networks as a novel tool for studying team ball sports as complex social systems. Journal of Science and Medicine in Sport. 2011;14(2):170–6. 10.1016/j.jsams.2010.10.459 21145787

[8] Boulding KE. General systems theory-the skeleton of science. Management science. 1956;2(3):197–208.

[9] Yamamoto Y, Yokoyama K. Common and unique network dynamics in football games. PloS one. 2011;6(12):e29638 10.1371/journal.pone.0029638 22216336 PMC3247158

[10] Wasserman S, Faust K. Social network analysis: Methods and applications: Cambridge university press; 1994.

[11] Whyte WF. Street corner society: the social structure of an Italian slum. 1943.

[12] Mitchell JC. Social networks in urban situations: analyses of personal relationships in Central African towns: Manchester University Press; 1969.

[13] Wäsche H, Dickson G, Woll A, Brandes U. Social network analysis in sport research: an emerging paradigm. European Journal for Sport and Society. 2017;14(2):138–65.

[14] Clemente FM, Martins FML, Kalamaras D, Mendes RS. Network analysis in basketball: inspecting the prominent players using centrality metrics. Journal of Physical Education and Sport. 2015;15(2):212.

[15] Duch J, Waitzman JS, Amaral LAN. Quantifying the performance of individual players in a team activity. PloS one. 2010;5(6):e10937 10.1371/journal.pone.0010937 20585387 PMC2886831

[16] Emirbayer M. Manifesto for a relational sociology. American journal of sociology. 1997;103(2):281–317.

[17] Elster J. Nuts and bolts for the social sciences: Cambridge University Press; 1989.

[18] Loureiro M, Hurst M, Valongo B, Nikolaidis P, Laporta L, Afonso J. A Comprehensive Mapping of High-Level Men’s Volleyball Gameplay through Social Network Analysis: Analysing Serve, Side-Out, Side-Out Transition and Transition. Montenegrin Journal of Sports Science and Medicine. 2017;6(2):35–41.

[19] Hurst M, Loureiro M, Valongo B, Laporta L, Nikolaidis P, Afonso J. Systemic Mapping of High-Level Women’s Volleyball using Social Network Analysis: The Case of Attack Coverage, Freeball and Downball. Montenegrin Journal of Sports Science and Medicine. 2017;6.

[20] Bonacich P, Lloyd P. Eigenvector-like measures of centrality for asymmetric relations. Social networks. 2001;23(3):191–201.

[21] Borgatti SP, Mehra A, Brass DJ, Labianca G. Network analysis in the social sciences. science. 2009;323(5916):892–5. 10.1126/science.1165821 19213908

[22] Loureiro M, Hurst M, Valongo B, Laporta L, Nikolaidis P, Afonso J. A comprehensive mapping of high-level men’s volleyball game through Social Network Analysis: Analyzing complexes 0, I, II and III. Montenegrin Journal of Sports Science and Medicine. in press.

[23] Hurst M, Loureiro M, Valongo B, Laporta L, Nikolaidis PT, Afonso J. Systemic Mapping of High-Level Women's Volleyball using Social Network Analysis: The Case of Serve (K0), Side-out (KI), Side-out Transition (KII) and Transition (KIII). International Journal of Performance Analysis in Sport. 2016;16(2).

[24] Sasaki K, Yamamoto T, Miyao M, Katsuta T, Kono I. Network centrality analysis to determine the tactical leader of a sports team. International Journal of Performance Analysis in Sport. 2017;17(6):822–31. 10.1080/24748668.2017.1402283

[25] Cotta C, Mora AM, Merelo JJ, Merelo-Molina C. A network analysis of the 2010 FIFA world cup champion team play. Journal of Systems Science and Complexity. 2013;26(1):21–42.

[26] Mesquita I, Palao J, Marcelino R, Afonso J. Performance analysis in indoor volleyball and beach volleyball. McGarry T, ODonoghue P, Sampaio J, eds Handbook of sports performance analysis London: Routledge 2013:367–79.

[27] Palao JM, Santos JA, Ureña A. Effect of the manner of spike execution on spike performance in volleyball. International Journal of Performance Analysis in Sport. 2007;7(2):126–38.

[28] Marcelino R, Afonso J, Moraes JC, Mesquita I. Determinants of Attack Players in High-Level Men's Volleyball. Kinesiology. 2014;46(2):234–41.

[29] Lobietti R, Cabrini P, Brunetti M. The side-out complex in volleyball: the effect of reception and attack performance with the final score. International Journal of Performance Analysis in Sport. 2009;9(3):390.

[30] Laporta L, Nikolaidis P, Thomas L, Afonso J. The Importance of Loosely Systematized Game Phases in Sports: The Case of Attack Coverage Systems in High-Level Women’s Volleyball. Montenegrin Journal of Sports Science and Medicine. 2015;4(1):19–24.

[31] Costa G, Afonso J, Brant E, Mesquita I. Differences in game patterns between male and female youth volleyball. Kinesiology. 2012;44(1):60–6.

[32] Fewell JH, Armbruster D, Ingraham J, Petersen A, Waters JS. Basketball teams as strategic networks. PloS one. 2012;7(11):e47445 10.1371/journal.pone.0047445 23139744 PMC3490980

[33] Grund TU. Network structure and team performance: The case of English Premier League soccer teams. Social Networks. 2012;34(4):682–90.

[34] Quiroga ME, García-Manso JM, Rodríguez-Ruiz D, Sarmiento S, De Saa Y, Moreno MP. Relation between in-game role and service characteristics in elite women's volleyball. The Journal of Strength & Conditioning Research. 2010;24(9):2316–21. 20703161 10.1519/JSC.0b013e3181e3812e

[35] Afonso J, Mesquita I. Determinants of block cohesiveness and attack efficacy in high-level women's volleyball. European Journal of Sport Science. 2011;11(1):69–75.

[36] Afonso J, Mesquita I. Pilot study on the attack tempo in women's volleyball [Estudo piloto acerca do tempo de ataque em voleibol feminino]. Revista Portuguesa de Ciências do Desporto. 2007;7(Suppl.1):47.

[37] Laporta L, Nikolaidis P, Thomas L, Afonso J. Attack Coverage in High-Level Men’s Volleyball: Organization on the Edge of Chaos? Journal of Human Kinetics. 2015;47/2015:249–57. 10.1515/hukin-2015-0080 26557208 PMC4633260

[38] Hileno R, Buscà B. OBSERVATIONAL TOOL FOR ANALYZING ATTACK COVERAGE IN VOLLEYBALL [Herramienta observacional para analizar la cobertura del ataque en voleibol]. Revista Internacional de Medicina y Ciencias de la Actividad Física y el Deporte. 2012;12(47):557–70.

[39] Selinger A, Ackermann-Blount J. Arie Selinger's Power Volleyball. New York: St.Martin's Press; 1986.

[40] Araújo R, Afonso J, Mesquita I. Procedural knowledge, decision-making and game performance analysis in Female Volleyball's attack according to the player's experience and competitive success. International Journal of Performance Analysis in Sport. 2011;11(1):1–13.

[41] Hughes M, Cooper S-M, Nevill A. Analysis procedures for non-parametric data from performance analysis. International Journal of Performance Analysis in Sport. 2002;2(1):6–20.

[42] Fleiss JL, Levin B, Paik MC. Statistical methods for rates and proportions: John Wiley & Sons; 2013.

[43] Tabachnick BG, Fidell LS. Using Multivariate Statistics. 5 ed. Boston: Pearson; 2007.

[44] Araújo R, Moraes C, Coutinho P, Mesquita I, editors. Determinants related to the attack tempo in high level male volleyball World Congress of Performance Analysis of Sport IX; 2012; Worcester: University of Worcester.

[45] Afonso J, Esteves F, Araújo R, Thomas L, Mesquita I. Tactical determinants of setting zone in elite men's volleyball. Journal of Sports Science and Medicine. 2012;11(1):64–70. 24149123 PMC3737848

[46] Castro J, Mesquita I. Analysis of the attack tempo determinants in volleyball's complex II—a study on elite male teams. International Journal of Performance Analysis in Sport. 2010;10(3):197–206.

[47] Afonso J, Mesquita I, Marcelino R, Silva JA. Analysis of the setter's tactical action in high-performance women's volleyball. Kinesiology. 2010;42(1):82–9.

[48] Garganta J. Trends of tactical performance analysis in team sports: bridging the gap between research, training and competition. Revista Portuguesa de Ciências do Desporto. 2009;9(1):81–9.

[49] Butts CT. A relational event framework for social action. Sociological Methodology. 2008;38(1):155–200.

[50] Clemente FM, Martins FML, Mendes RS. There are differences between centrality levels of volleyball players in different competitive levels? Journal of Physical Education and Sport. 2015;15(2):272.

[51] Marcelino R, Mesquita I, Sampaio J. Effects of quality of opposition and match status on technical and tactical performances in elite volleyball. Journal of Sports Sciences. 2011;29(7):733–41. 10.1080/02640414.2011.552516 21424980

[52] Estéve ÁV, Fernández-Echeverría C, González-Silva J, Claver F, Moreno MP. Variables that Predict Serve Efficacy in Elite Men’s Volleyball with Different Quality of Opposition Sets. Journal of human kinetics. 2018;61(1):167–77. 29599869 10.1515/hukin-2017-0119 PMC5873346

[53] Marcelino R, Sampaio J, Mesquita I. Attack and serve performances according to the match period and quality of opposition in elite volleyball matches. Journal of Strength and Conditioning Research. 2012;26(12):3385–91. 10.1519/JSC.0b013e3182474269 22207260

