# Supplementary material for: Interaction network analysis of the six game complexes in high-level volleyball through the use of Eigenvector Centrality
Source: PLoS One. 2018 Sep 11;13(9):e0203348. doi: 10.1371/journal.pone.0203348 (PMC6133287; doi:10.1371/journal.pone.0203348)
Supplement: S1 Table — (DOCX) [file pone.0203348.s001.docx]

**Table 1. Summary of variables and occurrence in the game**

| **Variable** | **Categories** | **Game complexes** |
| --- | --- | --- |
| Initial Position of the Server | Zones 1, 6 or 5. | K0 |
| Serve Type | Float jump serve, Jump serve, and Standing serve. | K0 |
| Zone of First Contact | Zones 1 to 6. | KI to KIII |
| Setting Conditions | A (ideal), B (intermediate), and C (out-of-system). | KI to KV |
| Attack Zone | Zones 1 to 6. | KI to KV |
| Attack Tempo | Synchronization between the setter and the attacker (Tempo 1, 2 and 3). | KI to KV |
| Block Opposition | No-Block (B0), Single Block (B1), Double Block (B2), Triple Block (B3). | KII and KIII |
| Number of Available Players Before Attack Coverage | 1 to 5. | KIV |
| Number of Coverage Lines | 1 to undetermined. | KIV |
| Freeball or Downball | FB or DB | KV |
| Target Zone in KV | Offensive zone (3-meter line to the net) or defensive zone (behind the 3-meter line). | KV |
