# Supplementary material for: Interaction network analysis of the six game complexes in high-level volleyball through the use of Eigenvector Centrality
Source: PLoS One. 2018 Sep 11;13(9):e0203348. doi: 10.1371/journal.pone.0203348 (PMC6133287; doi:10.1371/journal.pone.0203348)
Supplement: S2 Table — (DOCX) [file pone.0203348.s002.docx]

**Table 2. Eigenvector Centrality values for Complex 0:**

| **Serve Type** | **Jump** | 0.09 |
| --- | --- | --- |
|  | **Jump-Float** | 0.10 |
|  | **Standing float** | 0.08 |
| Initial Position of the Server | **Z1** | 0.10 |
|  | **Z6** | 0.09 |
|  | **Z5** | 0.09 |
