# Supplementary material for: Interaction network analysis of the six game complexes in high-level volleyball through the use of Eigenvector Centrality
Source: PLoS One. 2018 Sep 11;13(9):e0203348. doi: 10.1371/journal.pone.0203348 (PMC6133287; doi:10.1371/journal.pone.0203348)
Supplement: S3 Table — (DOCX) [file pone.0203348.s003.docx]

**Table 3. Eigenvector Centrality values for Complex I:**

| **First Contact (Reception zone)** | **Z1** | 0.32 |
| --- | --- | --- |
|  | **Z2** | 0.16 |
|  | **Z3** | 0.21 |
|  | **Z4** | 0.18 |
|  | **Z5** | 0.32 |
|  | **Z6** | 0.30 |
| **Setting Conditions** | **A** | 0.52 |
|  | **B** | 0.52 |
|  | **C** | 0.55 |
| **Attack Zone** | **Z1** | 0.37 |
|  | **Z2** | 0.54 |
|  | **Z3** | 0.48 |
|  | **Z4** | 0.54 |
|  | **Z5** | 0.29 |
|  | **Z6** | 0.38 |
| **Attack Tempo** | **1** | 0.37 |
|  | **2** | 0.46 |
|  | **3** | 0.46 |
