# Supplementary material for: Interaction network analysis of the six game complexes in high-level volleyball through the use of Eigenvector Centrality
Source: PLoS One. 2018 Sep 11;13(9):e0203348. doi: 10.1371/journal.pone.0203348 (PMC6133287; doi:10.1371/journal.pone.0203348)
Supplement: S4 Table — (DOCX) [file pone.0203348.s004.docx]

**Table 4. Eigenvector Centrality values for Complex II:**

| **Fist Contact (Defense zone)** | **Z1** | 0.52 |
| --- | --- | --- |
|  | **Z2** | 0.38 |
|  | **Z3** | 0.46 |
|  | **Z4** | 0.47 |
|  | **Z5** | 0.55 |
|  | **Z6** | 0.50 |
|  | **OT** | 0.36 |
| **Setting Conditions** | **A** | 0.51 |
|  | **B** | 0.47 |
|  | **C** | 0.65 |
| **Attack Zone** | **Z1** | 0.52 |
|  | **Z2** | 0.59 |
|  | **Z3** | 0.50 |
|  | **Z4** | 0.64 |
|  | **Z5** | 0.14 |
|  | **Z6** | 0.50 |
| **Attack Tempo** | **1** | 0.41 |
|  | **2** | 0.52 |
|  | **3** | 0.52 |
| **Block Opposition** | **No-block** | 0.42 |
|  | **Single** | 0.46 |
|  | **Double** | 0.45 |
|  | **Triple** | 0.39 |
