# Supplementary material for: Interaction network analysis of the six game complexes in high-level volleyball through the use of Eigenvector Centrality
Source: PLoS One. 2018 Sep 11;13(9):e0203348. doi: 10.1371/journal.pone.0203348 (PMC6133287; doi:10.1371/journal.pone.0203348)
Supplement: S6 Table — (DOCX) [file pone.0203348.s006.docx]

**Table 6. Eigenvector Centrality values for Complex IV:**

| **Setting Conditions** | **A** | 0.32 |
| --- | --- | --- |
|  | **B** | 0.31 |
|  | **C** | 0.48 |
| **Attack Zone** | **Z1** | 0.23 |
|  | **Z2** | 0.46 |
|  | **Z3** | 0.29 |
|  | **Z4** | 0.45 |
|  | **Z5** | 0.00 |
|  | **Z6** | 0.26 |
| **Attack Tempo** | **1** | 0.27 |
|  | **2** | 0.38 |
|  | **3** | 0.37 |
| **Number of attackers available before KIV** | **Two attackers** | 0.73 |
|  | **Three attackers** | 0.75 |
|  | **Four attackers** | 0.74 |
| **Number of coverage lines** | **One line** | 0.31 |
|  | **Two lines** | 0.80 |
|  | **Three lines** | 0.82 |
