# Supplementary material for: Interaction network analysis of the six game complexes in high-level volleyball through the use of Eigenvector Centrality
Source: PLoS One. 2018 Sep 11;13(9):e0203348. doi: 10.1371/journal.pone.0203348 (PMC6133287; doi:10.1371/journal.pone.0203348)
Supplement: S7 Table — (DOCX) [file pone.0203348.s007.docx]

**Table 7. Eigenvector Centrality values for Complex V:**

| **Setting Conditions** | **A** | 0.48 |
| --- | --- | --- |
|  | **B** | 0.38 |
|  | **C** | 0.31 |
| **Attack Zone** | **Z1** | 0.26 |
|  | **Z2** | 0.45 |
|  | **Z3** | 0.44 |
|  | **Z4** | 0.43 |
|  | **Z5** | 0.00 |
|  | **Z6** | 0.21 |
| **Attack Tempo** | **1** | 0.42 |
|  | **2** | 0.41 |
|  | **3** | 0.25 |
| **Freeball** | 1 | |
| **Downball** | 0.88 | |
| **Target Zone of KV** | **Defense zone** | 0.89 |
|  | **Attack zone** | 0.91 |
